# Meteorological Table

**Published:** 1859-12

**Authors:** J. G. Westmoreland

**Affiliations:** Smithsonian Institution, Oct., 1859, at Atlanta, Ga.


### METEOROLOGICAL OBSERVATIONS,

Taken by J. G. Westmoreland, M. D., for the Smithsonian Institution, Oct., 1859, at Atlanta, Ga., Latitude 33 deg. 45 min.—Longitude 7 deg. 30 min..—Height above the Sea, 1050 feet.

| Days of Mo. | BAROMETER. | | | THERMOMETER. | | | RAIN AND SNOW. | | CLOUDS. | | | WIND. | | |
|---|---|---|---|---|---|---|---|---|---|---|---|---|---|---|
| | 7 A. M. | 2 P. M. | 9 P. M. | 7 A. M. | 2 P. M. | 8 P. M. | amt.rn | In.sn'w | 7A. M. | 2 P. M. | 9 P. M. | 7A.M. | 2P.M. | 9P.M. |
| 1 | 30.18 | 30,20 | 30.15 | 55 | 68 | 62 | | | 10 | 10 | 10 | E | S | S |
| 2 | 30.12 | 30.18 | 30.10 | 54 | 70 | 60 | | | 5 | 5 | 5 | S W | S W | S W |
| 3 | 30.05 | 30.10 | 30.05 | 48 | 66 | 48 | 1.75 | | 5 | 5 | 5 | N | N E | S E |
| 4 | 20.05 | 30.05 | 30.10 | 48 | 48 | 48 | 25 | | 10 | 10 | 10 | S E | S E | S E |
| 5 | 30.10 | 30.15 | 30.15 | 50 | 52 | 56 | 1.50 | | 10 | 10 | 10 | S E | S E | S E |
| 6 | 30.10 | 30.00 | 29.95 | 60 | 64 | 60 | | | 10 | 5 | 5 | S E | S | S |
| 7 | 29.80 | 29.85 | 29.96 | 30 | 29 | 26 | 50 | | 10 | 5 | 5 | N W | N | N W |
| 8 | 30.00 | 30.05 | 30.12 | 16 | 26 | 26 | | | 0 | 0 | 0 | N W | N W | N W |
| 9 | 30.20 | 30.27 | 30.25 | 24 | 36 | 32 | | | 0 | 0 | 0 | N W | N W | N W |
| 10 | 30.20 | 30.20 | 30.15 | 30 | 40 | 36 | | | 0 | 0 | 0 | N W | N W | N W |
| 11 | 30.10 | 30.10 | 30.05 | 30 | 50 | 40 | | | 0 | 0 | 0 | N W | N W | N W |
| 12 | 29.95 | 29.95 | 29.90 | 32 | 55 | 40 | | | 0 | 0 | 0 | N W | N W | N W |
| 13 | 29.85 | 29.80 | 29.85 | 32 | 50 | 40 | | | 0 | 0 | 0 | N W | N W | N W |
| 14 | 29.90 | 29.90 | 29.95 | 32 | 50 | 44 | | | 0 | 0 | 0 | N W | N W | N W |
| 15 | 29.95 | 30.00 | 30.05 | 32 | 48 | 48 | | | 0 | 5 | 5 | N W | E | E |
| 16 | 29.93 | 29.80 | 29.90 | 40 | 40 | 38 | 1.00 | | 10 | 10 | 10 | E | E | E |
| 17 | 30.00 | 30.00 | 29.90 | 40 | 48 | 38 | | | 10 | 10 | 5 | W | W | N W |
| 18 | 29.80 | 29.95 | 29.95 | 32 | 48 | 40 | | | 0 | 0 | 5 | N W | N W | S E |
| 19 | 29.90 | 29.80 | 29.75 | 38 | 46 | 40 | | | 5 | 10 | 10 | S E | S E | S E |
| 20 | 29.85 | 29.85 | 29.85 | 34 | 34 | 32 | | | 5 | 5 | 5 | N W | N W | N W |
| 21 | 29.85 | 29.87 | 29.95 | 26 | 30 | 26 | | | 5 | 5 | 0 | N W | N | N W |
| 22 | 29.85 | 29.90 | 29.90 | 20 | 36 | 30 | | | 0 | 0 | 0 | N W | N W | N W |
| 23 | 29.80 | 29.88 | 29.90 | 16 | 32 | 26 | | | 0 | 0 | 0 | N W | N W | N W |
| 24 | 29.98 | 30.02 | 30.00 | 24 | 36 | 30 | | | 0 | 0 | 0 | N W | N W | N W |
| 25 | 30.00 | 30.10 | 30.05 | 26 | 48 | 46 | | | 0 | 0 | 5 | N W | N E | S |
| 26 | 30.05 | 30.08 | 30.05 | 48 | 60 | 56 | | | 5 | 5 | 5 | S W | S W | W |
| 27 | 30.10 | 30.08 | 30.05 | 54 | 56 | 54 | 1.00 | | 10 | 10 | 10 | S | S | S |
| 28 | 30.00 | 30.05 | 30.05 | 56 | 64 | 58 | | | 10 | 5 | 5 | S | S W | S W |
| 29 | 29.92 | 29.80 | 29.78 | 44 | 44 | 48 | 4.25 | | 10 | 10 | 10 | S | E | S |
| 30 | 29.80 | 29.80 | 29.85 | 48 | 44 | 40 | 25 | | 5 | 10 | 5 | S W | W | W |
| 31 | 29.80 | 29.85 | 29.90 | 32 | 32 | 28 | 50 | 4 | 10 | 10 | 10 | N W | NW | N W |

Explanation of Table.—0 Signifies Entire Clearness—5 Half Cloudy—10 Entire Cloudiness.

